# Factors Affecting the Outcome of Unstable Intertrochanteric Fractures Managed With Proximal Femoral Nail Anti-Rotation 2: A Prospective Outcome Study in Elderly Indian Population

**DOI:** 10.7759/cureus.11973

**Published:** 2020-12-08

**Authors:** Shakti Swaroop, Prateek Gupta, Rajesh Bawari, Sanjiv K Marya, Swati Patnaik

**Affiliations:** 1 Orthopedics, Institute of Medical Sciences, SUM Hospital, Siksha 'O' Anusandhan (Deemed to be) University, Bhubaneswar, IND; 2 Orthopedics, Max Super Specialty Hospital, Saket, Delhi, IND; 3 Public Health Dentistry, Institute of Dental Sciences, Siksha 'O' Anusandhan (Deemed to be) University, Bhubaneswar, IND

**Keywords:** pfna 2, unstable intertrochanteric fracture, hip fractures

## Abstract

Background

The proximal femoral nail anti-rotation Asia (PFNA 2) is an implant designed for unstable osteoporotic intertrochanteric fractures in Asians as the PFNA was designed for Caucasians and had various complications when applied to the Asian population due to the femoral geometrical mismatch. This study observes the functional outcomes and complications associated with PFNA 2 in unstable intertrochanteric fractures in the elderly Indian population.

Methods

Sixty-one above 60 years old patients with an unstable intertrochanteric fracture who were operated with PFNA 2 were included in this prospective observational study. They were followed up for one year. The functional and radiographic evaluations were done at 6, 12, 20 weeks, and the functional outcome was evaluated at the end of one year. Association of age, American Society of Anaesthesiologists (ASA) grade, AO Foundation classification, osteoporosis to the functional outcome of modified Harris hip score (MHHS) was evaluated.

Results

Type A2 fractures demonstrated a statistically higher-good reduction than Type A3 (Student t-test, P < 0.05). The difference in mean surgical duration in Type A3 (45.47 minutes) and Type A2 (40.30 minutes) was statistically significant (Student t-test, P < 0.05). Mean blood loss was 110.66 ml (SD = 48.40 ml). MHHS at 6, 12, 20 weeks, and one year were 40.37, 63.93, 79.03, and 82.34, respectively. At the end of the year, 46 (82.1%) patients achieved good scores, eight (14.3%) achieved fair scores, and two (3.5%) achieved poor scores. There was one case of nonunion and medial migration of the helical blade. The mortality rate was 6.55% at the end of one year.

Conclusion

A good reduction was associated with a better functional outcome. PFNA 2 is an efficient implant in managing unstable intertrochanteric fractures in elderly Indian patients with good outcomes, low morbidity rates, and mortality. Implant mismatch was not a problem in the Indian population. However, large multi-centric studies with a larger sample size are required. Moreover, achieving a good reduction cannot be over-emphasized in unstable intertrochanteric fractures, especially in the elderly, to achieve a good functional outcome.

## Introduction

Intertrochanteric hip fracture is a major public health problem in the geriatric age group. Definitive management is desired, considering age and associated co-morbidities. Indian population is at higher risk of osteoporosis and associated hip fractures [1]. A study revealed that 50% women and 36% men over the age of 50 have low bone mass in India [2]. Hip fractures are common in this group of population and 50% of hip fractures in elderly patients are intertrochanteric of which more than 50% are of unstable type [3]. Hip fractures are associated with the risk of urinary tract infections, pneumonia, bedsores, and thromboembolic complications. They cause physical impairment, reduce the quality of life, and cause significant mortality. Management of such fractures aims to achieve early union and mobilization of the patient where some form of internal fixation is the method of choice [4]. Commonly used internal fixation devices for intertrochanteric fractures are of two types: Sliding compression hip screw with side plate assembly, e.g., Dynamic Hip Screw (DHS) and intramedullary fixation devices. The compression hip screw is the standard implant in the management of stable inter-trochanteric fractures [5,6], but in unstable intertrochanteric fractures (AO Type 31A2 and Type 31A3) it has a higher incidence of cut-out failure (6% to 19%) [7,8]. However, an intramedullary device with a shorter lever is likely to improve the biomechanics providing more load sharing and limiting collapse at the fracture site [9].

The proximal femoral nail anti-rotation (PFNA), a modification of proximal femoral nail (PFN), was introduced in 2003, which features a helical blade. Biomechanical cadaveric studies demonstrated that PFNA fixation using a helical blade was better compared to the sliding hip screw. PFNA, characterized by rotational along with angular stability has biomechanically improved purchase in the osteoporotic bone due to the bony impaction it achieves in the femoral head and neck [10,11]. PFNA was designed for femoral geometric proportions of the Caucasian population but differences exist between Asian and Caucasian femoral geometry [12]. Serious complications occurred when PFNA was used for Asians [13] which led AO/ASIF to design a new proximal femoral nail anti-rotation Asia (PFNA 2) for Asian femoral geometry [14].

This study was undertaken to analyze the results of unstable intertrochanteric fracture of femur fixed with PFNA 2, its functional and radiological outcomes in the elderly Indian population. No previous studies have established the role of various factors affecting the outcome of unstable intertrochanteric fractures fixed with PFNA2 in the elderly Indian population with a prospective study.

## Materials and methods

The study was a prospective two-year study after approval from the scientific and ethical committee of the institution. Written informed consent was taken from all patients. Patients > 60 years with unilateral unstable inter-trochanteric fracture femur (Type 31A2, 31A3- AO classification), ambulatory prior to fracture, mentally sound and asymptomatic contra-lateral lower limb were included. Fractures with subtrochanteric extension, inflammatory arthritis, revision surgery, >2 weeks old fracture, active infection were excluded

Patient's demographic data, American Society of Anaesthesiologists (ASA) grading [15], mode of trauma, AO fracture classification, Neck shaft angle (NSA) of the normal hip NSA of the operated hip; and osteoporosis grading (Singh's index) [16] of the normal hip were recorded. Operative duration and intraoperative blood loss were noted. Fracture reduction was achieved on the fracture table and surgical fixation of the fracture done with PFNA II (Proximal Femoral Nail Anti-rotation Asia, Synthes, Switzerland). The accepted position of the blade intraoperatively was central or inferior in the anteroposterior (AP) view and central in the lateral view [17]. Immediate postoperative radiographs were evaluated for grading of the fracture reduction and measurement of Tip Apex Distance (TAD) as developed by Baumgaertner et al [18].The Helical blade position by dividing the femoral head into superior, central, and inferior thirds on the AP radiograph and anterior, central, and posterior thirds on the lateral radiograph as per Cleveland's zones [19,20]. The post-operative hip neck-shaft angle (NSA) to compare with the contralateral hip. Patients were allowed to weight-bear to pain tolerance with a walker subject to the general condition of the patient, intra-operative reduction, and bone quality. In patients with poor bone quality, comminution, or frail general condition weight-bearing was delayed. Besides postoperative radiographs, functional parameters were recorded as per the modified Harris hip score (MHHS) at 6, 12, 20 weeks, and 1 year. The MHHS score was graded as excellent (90-100), good (80-90), fair (70-80), and poor (<70). Radiological union and complications were recorded. Union was defined as bridging callus evident on three of the four cortices in two orthogonal radiographical views [20].

Statistical analysis was done using SPSS 21 (IBM Corp., Armonk, NY). Descriptive statistics were computed. Categorical variables were expressed as proportions and the annotations were assessed using Pearson's chi-square test for large frequencies and Fischer's exact test for small frequencies. T-tests were used for the significance of difference and the level of significance was assessed with P value (significant when P < 0.05). The MHHS was analyzed to find its association with age, AO fracture classification, reduction quality, Singh's osteoporotic index, and ASA grade as fixed variables and age as a covariate using the Analysis of Covariance (ANCOVA) test.

## Results

The total number of patients in our study were 61 which included 41 females (67.2 %) and 20 males (32.7 %). The mean age was 75.39 years (SD = 8.4years). 46 (75.4 %) fractures were of type 31A2 and 15 were of type 31A3 (AO Classification). 93% of patients were grade ≥3 as per Singh's index for osteoporosis, four patients had severe osteoporosis, i.e., grade <3. The mean duration from admission to surgery was 1.59 days (1-8 days). The ASA grading was grade 3 in 57.37% of patients. In 68.9% patients good reduction was achieved (type 31A2 - 76%, type 31A3 - 46.7 %). Type A2 fractures had a statistically higher number of good reductions (P < 0.05). The mean duration of surgery was 41.57 minutes (SD = 6.24 minutes). The difference between the mean surgical duration of Type A3 (45.47 minutes) and Type A2 fractures (40.30 minutes) was statistically significant (P < 0.05). Mean TAD was 21.72 mm. Mean TAD for type A2 fracture and type A3 fractures was 21.53 mm and 22.32 mm, respectively. No significant difference was observed between type 31A2 and 31A3 fractures. The type of fracture did not influence the TAD (P > 0.05). 86.8% of the patients had a TAD < 25 mm. Forty-nine out of 61 patients (80.3%) had the Helical blade placed in the center-center zone in the head and neck of the femur. Ten patients had a central-posterior position and one each had an inferior-central and central-anterior position. The mean blood loss was 110.66 ml (SD = 48.40 ml). No blood transfusion was necessary. No early complications related to the implant and no infections were observed. Contralateral femur NSA and post-operated hip NSA were measured, and the mean values were 134.41° (SD = 1.53) and 134.54° (SD = 1.66), respectively. We did not come across implant mismatch in our group of patients. See Table 1.

**Table 1:** Demographics and results TAD, Tip Apex Distance

	AO Type A2	AO Type A3	Total
Mode of injury (No. of patients)			
Road Traffic Accident (RTA)	0	6	6
Trivial fall (TF)	46	9	55
Intra op reduction (Baumgartner’s)			
Good	35	7	42
Acceptable	11	8	19
Singh’s Index of osteoporosis			
Grade ≥3	44	13	57
Grade <3	2	2	4
Mean duration of surgery (minutes)	40.30 (6.30)	45.47 (4.20)	41.57 (6.24)
TAD mean (mm)	21.53 (3.03)	22.32 (2.34)	21.72 (2.89)
Blood loss (ml)			
≥100 ml	35	15	50
<100 ml	11	0	11
Weight-bearing to tolerance mobilization			
<2 days	45	10	55
>4 weeks	0	5	5

The MHHS at 6, 12, 20 weeks, and 1 year were 40.37, 63.93, 79.03, and 82.34, respectively. At the end of one year, 46 (82.1%) patients had a good score, 8 (14.3%) had a fair score and only 2 (3.5%) had poor scores (Table 2).

**Table 2:** Modified Harris hip score to evaluate functional outcomes at 6 weeks, 12 weeks, 20 weeks and 1 year ASA, American Society of Anaesthesiologists

Modified Harris hip score (MHHS)								
Mean (Standard deviation)								
	AO Type		Reduction		ASA Grade			
Duration	A2	A3	Acceptable	Good	2	3	4	Total
Week 6	41.14	38.06	36.01	42.39	41.34	40.41	37.03	40.37
	(6.58)	(6.05)	(2.97)	(6.78)	(7.42)	(6.52)	(0.89)	(6.54)
Week 12	65.09	60.42	57.78	66.78	64.50	63.58	64.17	63.93
	(9.00)	(8.24)	(5.29)	(8.95)	(9.46)	(9.51)	(3.59)	(8.98)
Week 20	79.15	78.65	76.94	79.95	79.43	78.61	80.11	79.03
	(6.19)	(4.04)	(4.40)	(6.04)	(4.33)	(6.85)	(0.45)	(5.73)
1 year	82.72	81.06	80.75	83.03	83.90	81.43	82.50	82.34
	(5.21)	(4.29)	(5.02)	(4.93)	(3.46)	(5.88)	(2.95)	(5.03)

**Figure 1:**
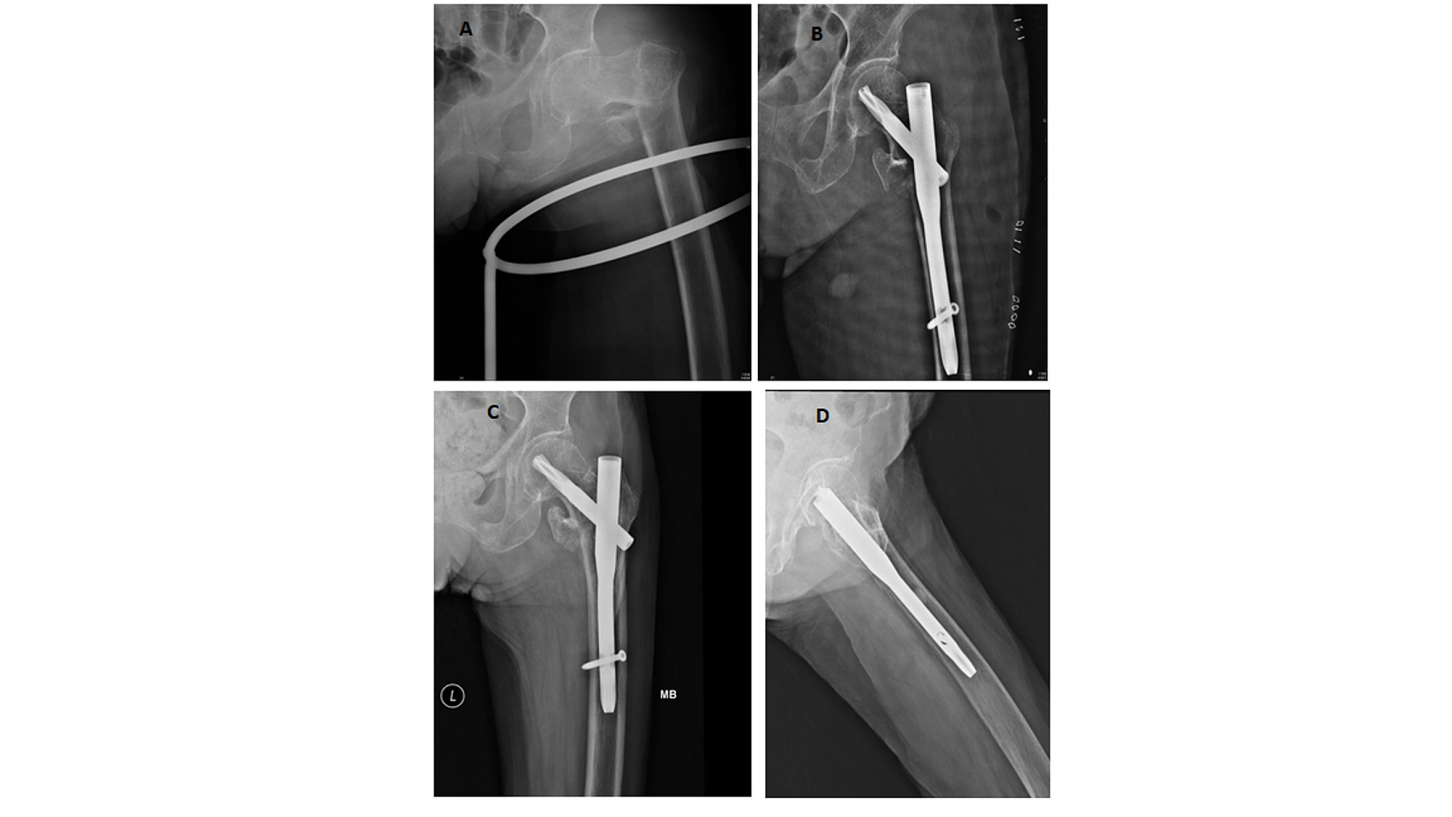
A: Patient with unstable intertrochanteric fracture of the femur; B: managed with PFNA2; C: fracture union in AP view radiograph: D: fracture union in lateral view radiograph PFNA 2, proximal femoral nail anti-rotation Asia; AP, anteroposterior

**Figure 2:**
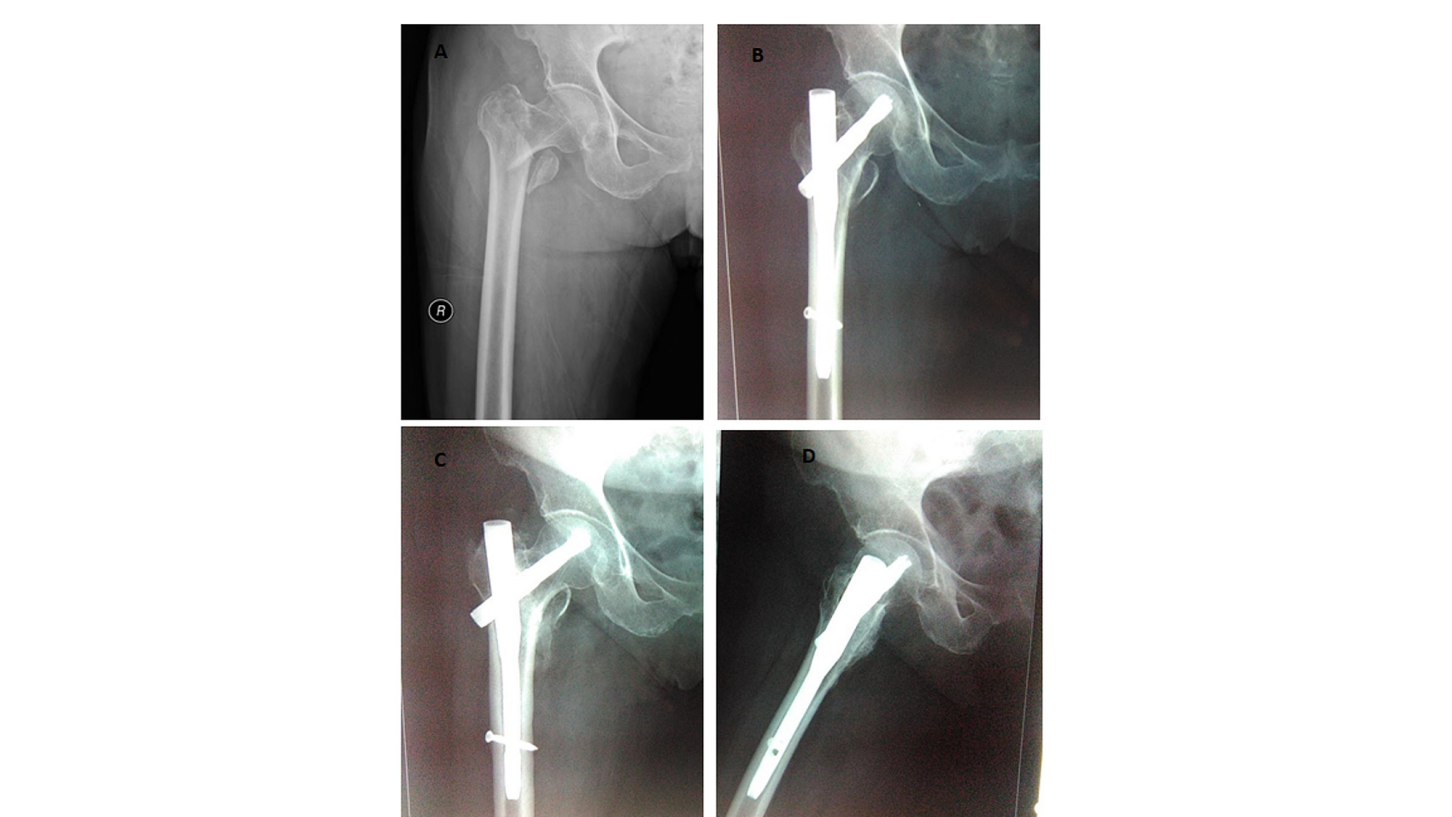
A: Patient with unstable intertrochanteric fracture; B: managed with PFNA 2; C: fracture union in AP view radiograph; D: fracture union in lateral view radiograph PFNA 2, proximal femoral nail anti-rotation Asia; AP, anteroposterior

Post-op radiographs showing unstable intertrochanteric fracture pattern, surgical fixation with PFNA2 and with fracture union achieved (Figures 1, 2).

## Discussion

Dynamic hip screw/sliding hip screw with plate is a commonly used implant for fixing inter-trochanteric fractures in elderly. In an in-vitro biomechanical study by Kuzyk et. al. compared extra-medullary to intra-medullary implants and found intra-medullary devices to be more efficient in achieving a stable fixation in unstable inter-trochanteric fractures. As an intra-medullary device is more medially placed in the medullary canal it has a shorter lever arm and shifts the load from posteromedial calcar towards the femoral axis which makes intramedullary nail a preferred implant in managing unstable inter-trochanteric fractures [21].

PFN an intramedullary device for intertrochanteric fractures has various advantages such as pre-reaming of the femoral canal is not necessary, higher rotatory stability due to an additional anti-rotational screw, and provision to lock the device dynamically or statically [22,23]. Complications that can occur in PFN are proximal screw cut out, difficulty in distal locking screw placement, and the Z-effect (i.e., Medial migration of the anti-rotation pin into the hip joint and the lateral displacement of the hip screw) [7,11]. Eventually, it fell out of popularity as new intramedullary devices were introduced. PFNA, an improved design over PFN was introduced by AO/ASIF has a major change, the helical blade with a gradually increasing diameter achieves impaction of the osteoporotic bone at the neck - head of the femur and facilitates anti-rotation at the fracture site [17,24]. PFNA had certain disadvantages in the Asian population like difficult entry of the nail, impingement at anterior cortex causing thigh pain or iatrogenic shaft fractures, and proud proximal end of the nail due to the variation in femoral anatomy. However, bending or breaking of the implant has not been reported [25,26]. AO/ASIF modified the PFNA design and introduced PFNA2 to prevent the complications arising from geometrical mismatch. The PFNA2 has a decrease in the mediolateral angle of the proximal nail from 6° to 5° (to reduce the risk of lateral cortex fracture). Secondly, the proximal part of the PFNA 2 is shortened to 45 mm and the end cap length is reduced to 2.5 mm (to reduce the incidence of hip pain). Thirdly, the lateral surface of the proximal end of PFNA 2 is flattened (to reduce the chance of fracture and loss of reduction during nail insertion) [26,27].

In our series of cases mean blood loss was 110.7 ml and no blood transfusion was required. PFNA 2 has significantly less blood loss compared to PFNA and DHS [13,28]. The mean duration of surgery was 41.57 minutes (SD = 6.24 minutes), comparable to the study by Li et al. [26]. The mean duration of surgery for type A2 fractures (40.30 min) was significantly less as compared to type A3 fractures (45.47 min) similar to Simmermacher et al. [24]. The mean duration of surgery was shorter as compared to fixation with other intramedullary nails, PFNA and surgical fixation with Dynamic/Compression hip screw devices [25,26,28] This may be due to better compatibility of PFNA 2 to Asian femoral anatomy. No cut-out of the helical blade was encountered, like in other studies [25,26]. TAD was <25 mm in 87% of the patients, as recommended by Baumgaertner et al. [18]. TAD < 25mm has been considered an important factor for the prevention of cut-out and successful osteosynthesis [18,29]. However, some authors have debated the impact of TAD on union, mechanical failure of the implant, or the functional outcome [30]. The center-center position of the helical blade has been considered to prevent the cut-out of the helical blade [18,29,30]

At the end of one year, 46 (82.1%) patients had a good MHH score. This is comparable to the study by Chiaoling et. al. with good to excellent results in 78.2% patients and by Ming et. al. who found good to excellent outcome in 81.9% patients [14,26]. A significant association of functional outcome score with the quality of primary reduction was observed. Better scores were associated with good reductions at both 6- and 12-week follow-up (P < 0.05). At 20 weeks and one-year good reduction had better functional scores; however, it was not statistically significant. Wei et. al. in a retrospective review of 204 patients reported good outcomes associated with good reduction [30]. No association of osteoporosis (Singh's Index) to the functional outcome was observed in our study. Wei et. al. suggested that the severity of osteoporosis did not influence the outcome in intertrochanteric fractures fixed with PFNA II [30]. All fractures united except one who had medial migration of the helical blade which was noted at 20 weeks post-surgery due to complaints of pain. However, nonunion was identified at the time of implant removal and underwent total hip replacement subsequently. Reports of medial migration of the helical blade have also been mentioned by Simmermacher et. al., Brunner et. al. and other authors [24-26]. 

Fracture of the femoral shaft or implant breakage was not observed which is attributed to the improved design of the distal end of the PFNA 2 with a flexible tip [14,17]. No patient in our study complained of hip or anterior thigh pain which may be due to a better fit of PFNA 2 in the Indian population. We did not notice any anterior impingement in our group of patients, which could cause the absence of thigh pain in our study. No intra-operative complication, post-operative infection, DVT, or embolism was reported. The functional outcome was not significantly associated with the type of fracture, ASA grade, or the age of the patient. Mortality rates in the elderly with inter-trochanteric fractures fixed with PFNA 2 have been reported as 7% [13]. Our mortality rate at 6.55% at one year was similar. Muller et. al. reported mortality rates of 26.7% (DHS) and 29.5% (PFNA) and had a higher mortality rate probably due to the higher mean age of their patients [29]. 

The limitation of our study is the absence of a control group to compare the functional outcome of unstable fractures fixed with other implants. A smaller sample size and no subjective method of measuring osteoporosis may be other drawbacks. We recommend such prospective studies with a larger sample size to gauge the functional outcome and complications associated with this implant using a better method to measure grade of osteoporosis. The message must go out to the young orthopedic surgeons that achieving a good reduction cannot be overemphasized even with a very advanced implant design.

## Conclusions

An intra-medullary device requiring minimally invasive procedure is the implant of choice in an unstable inter-trochanteric fracture in an osteoporotic elderly patient. It should allow a shorter duration of surgery with minimal blood loss. PFNA 2 fulfils these requirements. We recommend a good fracture reduction with the optimum position of the helical blade (center-center, in the two radiographic views) to achieve union and prevent cut out. TAD of < 25 mm is another important factor to prevent the cut-out of the helical blade. Osteoporosis, ASA grade, and type of fracture were not found to be associated with the functional outcome. A prospective study with functional outcomes of PFNA2 in unstable intertrochanteric fractures is lacking and this study should encourage orthopedic surgeons from India to further report larger groups in prospective studies which may help us further understand the implications and outcome of this implant in unstable fractures in the Indian population in long term.
